# Evolutionary success of the thrifty genotype depends on both behavioral adaptations and temporal variability in the food environment

**DOI:** 10.1038/s41598-023-33139-6

**Published:** 2023-05-17

**Authors:** Erasmo Batta, Christopher R. Stephens

**Affiliations:** 1 Posgrado en Ciencia e Ingeniería de la Computación, Universidad Nacional Autónoma de México, Mexico city, 04510 México; 2 Centro de Ciencias de la Complejidad, Universidad Nacional Autónoma de México, Mexico city, 04510 México; 3 Instituto de Ciencias Nucleares, Universidad Nacional Autónoma de México, Mexico city, 04510 México

**Keywords:** Computational science, Human behaviour, Risk factors, Behavioural ecology, Behavioural genetics

## Abstract

Obesity is a result of a long-term energy imbalance due to decisions associated with energy intake and expenditure. Those decisions fit the definition of heuristics: cognitive processes with a rapid and effortless implementation which can be very effective in dealing with scenarios that threaten an organism’s viability. We study the implementation and evaluation of heuristics, and their associated actions, using agent-based simulations in environments where the distribution and degree of richness of energetic resources is varied in space and time. Artificial agents utilize foraging strategies, combining movement, active perception, and consumption, while also actively modifying their capacity to store energy—a “thrifty gene” effect—based on three different heuristics. We show that the selective advantage associated with higher energy storage capacity depends on both the agent’s foraging strategy and heuristic, as well as being sensitive to the distribution of resources, with the existence and duration of periods of food abundance and scarcity being crucial. We conclude that a ”thrifty genotype” is only beneficial in the presence of behavioral adaptations that encourage overconsumption and sedentariness, as well as seasonality and uncertainty in the food distribution.

## Introduction

The current obesity epidemic represents one of the world’s most challenging public health problems. According to the World Health Organization^1^, in 2016 more than 1.9 billion adults were considered overweight and, of these, more than 650 million were classified as obese. Obesity is associated with an increase in both general and specific-disease related mortality^2,3^. Fat accumulation associated with obesity is due to a long-term positive energy imbalance, where energy intake is greater than energy expenditure, and is attributed to the interaction between an individual and an obesogenic environment. Modeling this phenomenon is exceedingly difficult due to the myriad factors involved. Moreover, each factor relates to others at multiple levels, thereby creating complex feedback loops. Although reductionist approaches have been the dominant framework for studying obesity, more systems-based, or complexity-based, papers on obesity have appeared^4–7^.

A recurring question in obesity research is: what has led to an explosion in obesity incidence in the last few decades? Have certain environmental factors changed radically? If so, which? Have our decision making processes changed? Both? One interesting line of research has been that associated with the notion that we are genetically maladapted to the current obesogenic environment relative to the environment in which our species originated. In particular, the “thrifty” genotype hypothesis^8,9^ is based on the idea that our evolution, in an environment where food resources were scarce, favoured those genes that allowed for better fat storage. However, within this “genomic” approach, although there is ample evidence that certain genetic polymorphisms can lead to an altered physiology that favours fat storage, these alterations are relatively rare and cannot be used to explain a phenomenon as universal as the current obesity epidemic. At heart, obesity, seen as a consequence of energy imbalance, is a result of human behavior—principally overeating and sedentariness—that are associated with individual decisions—eat/don’t eat, forage/don’t forage etc. Furthermore, these decisions are affected by a multitude of factors beyond those that might be associated with a microscopic “ome”, such as the genome, proteome or transcriptome. Indeed, it has been argued that what is required is a “Conductome”^10^, thought of as the universe of factors that influence a particular behaviour, such as overeating.

Decision-making theories based on utility function maximization are frequently inconsistent with human behaviour. The term *“cognitive bias”* was used in^11^ to refer to these discrepancies. A common characteristic of all these situations is attribute substitution, i.e*.*, the exchange of a computationally complex problem for a simpler one. One of the main criticisms of this description is that it just considers *cognitive bias* to be an inferior, secondary alternative due to a lack of sufficient computing resources^12–14^. A number of studies have found, however, that in particular contexts this kind of decision-making process generates better results than purely utility-based decision making^12,15,16^. This concept, which we shall refer to as *heuristic*, provides an explanation of efficient and immediate responses to risk scenarios.

Heuristics are particularly important in situations where survival is at risk and there is insufficient time to perform a detailed deliberation, or in states of cognitive depletion. These scenarios are not exclusive to human decision-making but are common to many living organisms. Many animal behaviours that fulfil simple decision rules can be viewed as heuristics^17^. Eating tends to involve more heuristic judgment than most behaviours, due to its intimate relationship with survival^18–21^. It is the adaptive match between cognitive and ecological structures that constitutes the basis of heuristic formation^13^. Heuristics are a natural consequence of certain, key environmental properties: Uncertainty, redundancy, finite sample size and variability in the relevance of environmental features. Final decisions are related to the environmental availability of energetic resources and the constraints on the execution of a specific physical activity. The environment provides a distribution of food resources with a particular energy density in space and time and an energy cost associated with their localization and consumption. The sensorial perception of the characteristics of a food resource depends on the complexity of the consuming organism^22–25^.

An organism’s learned experience in a given food environment allows it to label it as abundant or scarce in food resources for example. So, a food rich environment may provide enough resources such that consumption is only linked to physiological indicators of internal low energy states, whereas food scarcity may induce consumption beyond satiety so as to accumulate reserves. Uncertainty in this sense is a powerful motive for the development of heuristics and other cognitive capabilities, as, for example, in the case of metacognitive judgments that have been observed in humans and animals^26–29^.

Accepting that obesity arises from obesogenic behaviors in a potentially obesogenic environment, and given the tremendous multi-factoriality of the problem, with heterogeneous, dynamic and adaptive risk factors, that range from the genetic to the social, an agent-based modeling approach offers several advantages relative to purely empirical approaches, or more standard mathematical modelling, not least of which is the possibility to compare and contrast behaviours in different environments. In this paper an agent-based model (ABM) is presented in order to simulate decision making in different food environments and show under what circumstances heuristic-environment interactions can lead to persistent energy imbalances.

The flexibility and heterogeneity of ABMs makes them a suitable testing ground for studies of cognition and decision making. Their *bottom up* approach makes it possible to see how particular microscopic properties can lead to emergent macroscopic regularities^30,31^. Various approaches have been proposed to model decision-making processes in ABMs using purely reactive agents with *if-then* rules that are inspired by psychological and neurological architectures^32^. Jansen and Jager proposed a decision architecture for agents that explicitly addresses heuristics: the *CONSUMAT* model^33^, which attempts to unify psychological theories of learning and satisfaction. The *CONSUMAT* heuristics are characterized by both the amount of cognitive effort required (as in dual approaches) as well as the degree of uncertainty associated with information gathered from the environment.

In summary: The aim of the present work is to use an ABM to analyse under what circumstances a thrifty genotype, as a potentially important causative factor in the present obesity epidemic, might be selected for. In particular, the goal is to understand whether and how its utility depends on the following factors: (1) behavioral adaptations, such as overconsumption and sedentariness; (2) the distribution in space and time of food resources; and (3) the application of different heuristics in decison making.

## Methods

### Agent model

The present ABM aims to test how different degrees of uncertainty in the environmental availability of energy sources, as well as different foraging strategies and associated heuristics, can promote a preference for higher energy storage capacity.

#### Agent metabolism

In this model, an agent, $\alpha$, monitors its energy, $E_{\alpha }(t)$, which is taken as a proxy for the agent’s body mass and is degraded according to two factors: intentional activities and basal metabolism. Base Metabolic Rate (BMR) is modeled as a constant rate, $M_b$, which represents energy used per unit mass per unit time for basic metabolic functions multiplied by the agent’s energy when it is above a threshold, $E_T$, in analogy with body-mass dependence in well known formulas for BMR, such as the Mifflin-St. Jeor equation^34^. As similar metabolic rates have been reported for human populations with radically different lifestyles^35^, we selected $M_b$ to be the same for every agent to make BMR proportional to only the “size” of the agent. However, below $E_T$, we consider the total energy expenditure per unit time as constant, to avoid the existence of agents that live indefinitely through a smaller and smaller base metabolism. Thus, the basal expenditure is $E_{\alpha }(t) M_b$ when $E_{\alpha }(t) > E_T$ and $E_T M_b$ otherwise. $E_T M_b$ represents the minimum amount of energy needed to keep an organism alive. Hence, $E_T$ should not be considered as the dividing line between healthy and unhealthy levels of energy reserves. Above $E_T$, the linear metabolic expenditure creates an upper bound on the energy that agents may accumulate for a given value of $M_b$, given a certain energy consumption, $E_s$, in a given time period. When the agent’s metabolic expenditure is equal to the amount of energy that can be consumed, the change in internal energy, $\Delta E_{\alpha }(t)$, of the agent due to consumption is zero. Hence, $\Delta E_{\alpha }(t) = E_s - M_b E_{\alpha }^{(max)} = 0$ and then $E_{\alpha }^{(max)} = \frac{E_s}{M_b}$.

In order to make contact with thrifty gene-type arguments, we also explore the role of different energy storage levels. Specifically, we consider agents with modifiable energy accumulation levels, $L_\alpha (t)$, associated with the “maximum” amount of energy they may store, viewed as a proxy for fat accumulation, given that the latter may be realized by multiple physiological mechanisms and at different scales, from the tissue and organ level to the cellular and sub-cellular level. From a thrifty gene perspective we consider this as a key parameter associated with an agent’s overall survival strategy and which may change in time according to the heuristic used by the agent. Note that we do not take $L_\alpha (t)$ to be a physiologically imposed upper limit on fat accumulation but, rather, use it to compare the relative advantage of agents associated with one level versus another. For instance, an interesting level is that associated with what is considered to be normal. For humans this is 5–20% for males and 20–30% for females^36^, while for our primate relatives, such as bonobos or baboons 1–2%^37^ are considered normal. For marine mammals in higher latitudes it can be as high as 50% or more. A question of interest is then: under what circumstances is a level of 2% versus 20% advantageous? We will argue that this normal level is a balance between the advantages of being able to survive food scarcity periods and the disadvantages of excess adiposity, such as reduced foraging capacity or being subject to higher predation rates.

#### Agent actions

We model an agent in a given food environment by means of a limited set of available intentional actions: (1) they can decide to eat or not eat all the food at a given position at any specific moment; (2) they may decide to expand their perception of the existence of food at their current location to also include any adjacent cells and/or; (3) they can decide to move or not to one of the eight closest contiguous spatial positions. Perception is taken to have an associated energy cost, $C_p$, which we take to be constant per unit time. Similarly, the cost of movement is modelled as the agent’s energy multiplied by a factor, $C_m$, to reflect the fact that moving more mass requires more energy. Additionally, we model the fact that a mass excess or deficit may inhibit movement by introducing an energy dependence to the probability for movement, *p*, as seen in Equation (1). The intuition behind this is two-fold: (1) bigger reservoirs of energy are more difficult to move, independently of whether or not the associated energy is considered healthy or not; and (2) organisms with very low energy experience some malfunctioning of their motor capacities. We choose the corresponding energy range of uninhibited movement to be between $E_s$, where $E_s$ is the “portion size” of energy potentially available in a given cell, and $E_T$. Hence, once an agent decides to move, the probability of doing so is the following:

1 $$\begin{aligned} p = {\left\{ \begin{array}{ll} \exp {(E_{\alpha }(t)-E_s)} &{}\text {if}\, E_{\alpha }(t)<E_s\\ \exp {(E_T-E_{\alpha }(t))}&{}\text {if}\, E_{\alpha }(t)>E_T\\ 1 &{}\text {otherwise.}\\ \end{array}\right. } \end{aligned}$$

Agents with an internal energy $E_{\alpha }(t)$, between $E_s$ and $E_T$, will move every time they decide to do so, while those that have an energy bigger than $E_T$, or smaller than $E_s$, will, on average, move less.

**Table 1:** Relevant action strategies and heuristic pseudo-codes for agents.

Strategy	Symbol	Eat	Perceive	Move
Static	S	1	0	0
Random foraging	RF	1	0	1
Directed foraging	DF	1	1	1
Feedback foraging	FF	1	1	1 if food is found,
				0 otherwise

Heuristic	Pseudo-code			
Repetition	$L_{\alpha }(t+1) = L_{\alpha }(t)$			
Imitation	Select a random agent, $\beta$, from current $\alpha$’s community then $L_{\alpha }(t+1) = L_{\beta }(t)$			
Inquiring	Select that agent, $\beta$, from current $\alpha$’s community with the biggest internal energy then $L_{\alpha }(t+1) = L_{\beta }(t)$			

When strategy is 1 or 0 means that the corresponding action is executed or not, respectively. Pseudo-code represents heuristics used to select an energy accumulation level ($L_{\alpha }$) at time *t* according to the heuristics of the CONSUMAT model. Agent $\alpha$ considers other agents within its community if they are in those cells that agent $\alpha$ can perceive.

A combination of the actions of eat-perceive-move we may think of as representing the *foraging strategy* of the organism. Although there are eight possible combinations of the vector of decisions (decision to eat, to perceive and to move) only a subset lead to interesting interactions with the environment and which are defined in Table (1): *Static* (S), which corresponds to an agent that passively waits for food in a given spatial position; *Random Foraging* (RF), which corresponds to a “Brownian” agent that forages randomly every time step; and *Directed Foraging* (DF), where an agent looks for food (perception) in adjacent cells and moves randomly to any cell that has food. If no food is perceived the agent moves to a neighboring cell chosen at random anyway. We also introduce a strategy, *Feedback Foraging* (FF), where after perception, if no food is located the agent can decide to stay put and not move. Thus, DF and FF are chosen to potentially differentiate between two distinct behaviors when confronted with a lack of food in the immediate vicinity—a sedentary behavior—stay put—with an active behavior—move in the hope that eventually food will be found.

We assume that the first decision an agent makes is about the consumption of food, followed by perception and then movement. Each strategy has an associated energy cost, depending on whether perception and/or movement are included. In conjunction with the base metabolism of the agent, this leads to a net total energy expenditure at a given time step. If an agent consumes food and its energy expenditure is less than $E_s$, then there is a positive energy imbalance in that time step. In the case that the agent does not consume, or that $E_s$ is less than the total energy expenditure, then there is a negative energy imbalance. We may characterise an environment that is *scarce* in food resources as being such that there is a persistent negative energy imbalance, which could, eventually, compromise the agent’s survival. On the contrary, we may characterise an environment as being *abundant* in food resources if there is a persistent positive energy imbalance.

#### Environmental conditions

The environment in which the agents’ strategies are enacted is modelled as a square lattice of spatial cells with periodic boundary conditions, wherein agents occupy only a single cell at a time, but where a cell can accommodate more than one agent. As mentioned above, units of energy (food resources), $E_s$, equivalent to “portion size”, are situated at each cell of the lattice, where each unit can be consumed in its entirety by only one agent at a time. An agent may attempt to eat, but without success, due either to competition or a lack of food in its cell.

Consumed resources are regenerated in a given cell with probability $p_g$ per unit time, which can be set as a constant parameter for every cell, or can be time varying, so as to mimic, for example, the effect of seasonal variation in food availability. This kind of uncertainty, which is a feature of the experiences of many “traditional” human societies^38,39^, has been used to provide a logic for the evolutionary utility of “thrifty” genotypes^40,41^. We will consider the periods of such “feast and famine” episodes to be chosen from a normal distribution with a particular mean ($t_{ab}$ and $t_{fam}$) and standard deviation (STD). We choose this particular set of features to model some degree of uncertainty in the availability of food resources and the regulation of energy which can generate an ecological pressure that favours certain foraging and energy accumulation strategies, or can cause organisms to restructure their behavior, so as to try different or new heuristics in their decision making. The magnitude and lengths of the periods of abundance and scarcity play a critical role in imposing selection pressure on the strategies and heuristics and, in particular, on the energy accumulation levels.

We can develop a quantitative idea of the potential impact of a period of abundance or scarcity of a given duration by considering how much energy an agent may accumulate/lose during a period of abundance/scarcity. For example, consider an agent with the least costly metabolic settings (which implies an S strategy) that consumes food every time step in the period of abundance, having started from zero energy. As there are two distinct energy accumulation regimes, $E_\alpha <E_T$ and $E_\alpha >E_T$, in the former, the agent has an energy gain $E_s-M_b E_T$ per time step, while, in the latter, the energy gain per time step is $E_s - M_b E_{\alpha }(t)$. Considering an initial energy state where $E_{\alpha }= E_T$, as the agent consumes food at every time step, the change in energy is such that $E_{\alpha }(t) = E_{\alpha }(t-1) + E_s - M_b E_{\alpha }(t-1)$. As $\Vert 1-M_b\Vert < 1$ we have that $E_\alpha (t) = \frac{E_s}{M_b} \left( 1 - (1 - M_b)^t \right) + (1 - M_b)^t E_T$. In order to compare different energy storage capacities in a simple way we take the energy accumulation level $L_\alpha (t)$ as a maximum energy the agent can reach if it is less than the energy maximum limit, $E_\alpha ^{(max)}$, where metabolic energy expenditure exceeds consumption. Again, we emphasize, that $L_\alpha (t)$ does not necessarily represent a physiological bound. With either of these limits we may determine how long a period of abundance would have to last in order to reach a given energy $E_\alpha (t)$,

2 $$\begin{aligned} t^{(E_T \rightarrow E_{\alpha })} = \frac{\log ( \frac{E_s}{M_b}-E_{\alpha }) - \log ( \frac{E_s}{M_b}-E_T)}{\log (1 - M_b)} \end{aligned}$$

   If we take as initial condition $E_\alpha = 0$, then we just need to add in the time to get from energy 0 to energy $E_T$, which is $t^{(0 \rightarrow E_T)}=E_T/(E_s-M_b E_T)$ yielding a total time $t^{(0 \rightarrow E_{\alpha })} = t^{(0 \rightarrow E_T)} + t^{(E_T \rightarrow E_{\alpha })}$.

On the other hand, we can also calculate how long a famine with no food availability should last in order to produce the metabolic death of an agent. For an agent that has the least costly metabolic settings, the time for the agent to exhaust their energy during a period without food regeneration is given by $t^{(E_{\alpha } \rightarrow 0)} = t^{(E_{\alpha } \rightarrow E_T)} + t^{(E_T \rightarrow 0)}$,

3 $$\begin{aligned} t^{(E_{\alpha } \rightarrow 0)} = \frac{\log (E_T) - \log (E_{\alpha })}{\log (1-M_b)} + \frac{1}{M_b} \end{aligned}$$

   The above logic will be useful for understanding under what conditions an agent with a given energy accumulation level may be expected to have a competitive advantage relative to one with a lower level, both in the capacity to accumulate energy as well as to survive a famine. For example, if the period of abundance of energy is not sufficiently long for an agent with a higher accumulation level to accumulate more energy than an agent with a lower level then the higher level is clearly of no use. This analysis is used to approximate the values $t_{ab}$ and $t_{fam}$ in the second set of simulations.

#### Heuristics and decision dynamics

Besides a foraging strategy and an energy accumulation level, we consider that each agent may also use a heuristic/decision rule, loosely aligned with the CONSUMAT schema, with the corresponding algorithms described in Table 1. Note that although in the CONSUMAT model different heuristics correspond to different types of decision problems, here we consider only one overall decision problem—how best to find food in an uncertain environment. We restrict the heuristics to be applied to the energy accumulation levels only, returning to their application to the different foraging strategies in a future paper. The three heuristics we consider are: Repetition, imitation and inquiring^42^. Repetition is the decision process that is the simplest to implement, as it only requires that an agent keep doing the same thing. The imitation or inquiring heuristics require a “social” component, where at each time step an agent $\alpha$ creates a pool of possible alternatives (other possible energy accumulation levels) by observing those agents that they can “perceive” in their community, $\alpha '$. In the case that the agent has no explicit perception element in their strategy, then the set $\alpha '$ is restricted to those strategies or accumulation levels present in agents located at the same position as the agent $\alpha$. On the contrary, when perception is an element in the agent’s strategy, then the set $\alpha '$ is extended to those strategies or accumulation levels that are also located in adjacent cells. For imitation, the probability to choose a particular energy accumulation level depends on the number of agents with that trait within the community (i.e., those agents in the cell where the agent is situated, and including the eight adjacent cells if extended perception is decided), as the selection of an agent to copy is done randomly, while, for inquiring, they select randomly an accumulation level from among those agents with maximum internal energy in the group they perceive.

Uncertainty in the decision-making process, as described in our model, arises from several sources. Obviously, uncertainty in the distribution of food resources is an important source. There is also uncertainty as to whether an agent will be able to move at all. In terms of the social component, there is uncertainty in calculating the utility of the strategies associated with the community of a given agent relative to the set of all strategies. In other words, an agent does not a priori know if the best strategy in their community is the best possible strategy overall. It is important to emphasize that, given that this is a competitive system, even if an agent chooses the optimal strategy, there is uncertainty as to the outcome, as a competitor may already have consumed the desired resource.

### Simulations

Agent systems were simulated with NetLogo 6.0.1^43^. The environment consisted of a square grid of 41$\times$41 cells with periodic boundary conditions. The initial agent population was 1680 agents chosen to be close to a density of one agent per cell. Each agent begins with an initial energy of 2 units. The energy limit that determines the probability of movement was set to $E_T=20$ and $M_b$, $C_m$ and $C_p$, were fixed at values 0.05, 0.02 and 0.01 respectively. These chosen values were products of a limited exploration of the range of parameters, where we search for evident effects from changes in accumulation levels and in the probability of regeneration. Thirty simulations were performed for each combination of parameters.

As with any ABM, there is a potentially large parameter space to be analysed. In the present study, as well as four foraging strategies and three heuristics, there are several parameters that can be varied, as discussed above. Additionally, the spatio-temporal distribution of $p_g$ also offers a rich source of variability for determining the relative advantages of one set of agent characteristics versus another. Below, we discuss only a subset of experiments and their results that represent what we believe to be the most important conclusions for understanding the possible origins of the obesity epidemic and its relation to the thrifty genotype.

The first sets of experiments were designed to better understand under what environmental circumstances the capacity to accumulate energy (fat) was advantageous, this being related to the “thrifty gene” logic. As the capacity to accumulate energy can be argued to be present in other organisms than humans, we first used only the heuristic “repetition”. In the first set of experiments, we compared different accumulation levels between 5 and 105 units in the context of the different foraging strategies and followed their evolution for 500 time steps. The environmental parameter $E_s$ was set to $E_s = 2$. In every simulation, agents were randomly initialized with one of two accumulation levels, corresponding to 840 agents of each level in the initial population. One of them was chosen to be 5 and the second was chosen to be one of 6, 15, 55 and 105 leading to differences in the energy accumulation levels between the two agent groups of 1, 10, 50 and 100 respectively. Note that by considering different accumulation levels we are effectively testing just how “thrifty” the thrifty genotype has to be in order to confer an advantage. Indeed, this poses the question of what a non-thrifty genotype looks like? We claim this would be an accumulation level adjusted to give net energy imbalance zero on average. Here, we take accumulation level 5 as the least thrifty, basal genotype with the others being progressively more thrifty. The environmental parameter $p_g$ was taken to be constant in time and varied from 0.1 (scarcity) to 1.0 (abundance) in intervals of 0.1, and then from 0.01 (extreme scarcity) to 0.15 in intervals of 0.01. In all these cases the environmental parameter $p_g$ was constant.

In a second set of simulations, $p_g$ was allowed to change in order to mimic the effects of seasonal changes in food availability, and modeled by periods of relative abundance (when $p_g=1$) and periods of relative scarcity (when $p_g=0$). The periods of abundance and scarcity were chosen from normal distributions ${\mathscr {N}}(t_{ab},STD)$ and ${\mathscr {N}}(t_{fam},STD)$, where $t_{ab}=60$ time steps for the abundance and $t_{fam}=40$ for the period of scarcity. In this context, a “cycle” is the occurrence of a period of relative abundance, followed by a period of relative scarcity. These values were chosen using our analysis in Eqs. (2) and (3) where $t^{(0 \rightarrow E_{\alpha })} = 50.54 < 60$ and $t^{(E_{\alpha } \rightarrow 0)} = 39.72 < 40$ when $E_s = 3.0$ in (3). By varying STD we could introduce different degrees of uncertainty into the seasonal availability of food resources. We considered STD = 0 (constant periods), 1 and 5. System development was followed for 1000 time steps, with data collected by population for each action, decision rule and accumulation level. In these particular simulations, agents could have one of two accumulation levels: 55 and 105. Meanwhile, the amount of food in every cell was increased from $E_s = 2.0$ to 3.0, so as to make the maximum level of energy, $E_{\alpha }^{(max)}= 60$, take a value between 55 and 105. In these experiments we also considered heuristics other than repetition, the consequence being that an agent could potentially change its energy accumulation strategy over time by imitating the strategy of another agent, or by deducing the existence of a better strategy from concurrent agents (inquiring).

In the final set of experiments we analysed how varying the initial population affected the results of the second set of experiments in the case of STD = 0. Initial populations of 1680, 1260, 840 and 420 were considered and randomly spread onto the grid thus representing approximately uniform initial densities of agents per cell of 1.0, 0.75, 0.5 and 0.25, respectively. A summary of the parameters used in simulations can be found in Table 2.

**Table 2:** Summary of parameter values and symbols employed in the ABM experiments.

Symbol	Parameter	EXP 1	EXP 2	EXP 3
$E_{\alpha }(t)$	Internal energy of agent $\alpha$ at time *t*			
$E_T$	Energy threshold between metabolic			
	expenditure/movement regimes	20	20	20
$M_b$	Base metabolic rate	0.05	0.05	0.05
$C_p$	Energetic cost of active perception	0.01	0.01	0.01
$C_m$	Energetic cost of movement	0.02	0.02	0.02
*p*	Probability of movement			
*S*, *RF*, *SF*, *FF*	Foraging strategies			
$L_{\alpha }(t)$	Energy accumulation level of $\alpha$ at time *t*	5, 6, 15, 55, 105	55, 105	55, 105
$E_s$	Amount of energy at each grid cell	2.0	3.0	3.0
$p_g$	Probability of regeneration of food resource	[0.1, 1.0]	0.0, 1.0	0.0, 1.0
	per cell per unit time			
$t^{(0 \rightarrow E_{\alpha })}$	Time required by S agents to pass from		50.54	50.54
	zero to a given value of energy (57) with			
	abundance of food resources			
$t_{ab}$	Mean duration of abundance periods		60	60
$t^{(E_{\alpha } \rightarrow 0)}$	Time required by S agents to pass from a		39.72	39.72
	a given value of energy (55) to zero with no			
	food resources			
$t_{fam}$	Mean duration of famine periods		40	40
	Number of cycles		10	10
	Initial population of agents	1680	1680	1680, 1260, 840, 420

## Results

**Figure 1:**
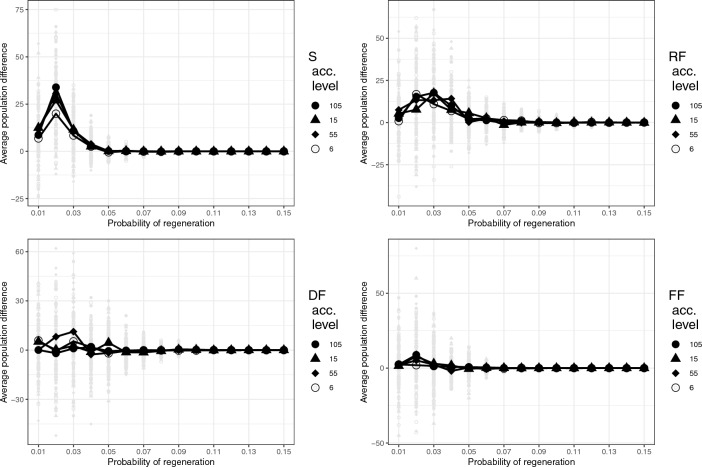
Difference in population size of competing agents with different energy accumulation levels, where agents repeat the same strategy: (Top, left:) S, (Top, right:) RF, (Bottom, left:) DF and (Bottom, right:) FF, for 500 time steps in an environment with a constant value of $p_g$ between 0.01 and 0.15. $50\%$ of the agents start with an energy level of 5 and $50\%$ start with an energy level of 6, 15, 55 or 105 respectively. Graphs show the average of 30 repetitions using the same parameters. The gray points in the background represent the result for every experiment.

The results of the first set of experiments can be seen in Fig. 1. There, the vertical axis represents the difference in the number of agents present in the population after 500 time steps with differing accumulation levels versus agents with a level of 5. Thus, in the top left figure we see a slight advantage for higher accumulation levels in the case of strategy S (where agents eat, do not perceive and do not move) for $p \sim [0.01-0.04]$, with there being approximately 20–30 agents more with accumulation levels $>5$ than with accumulation level 5. This relative advantage of a “thrifty gene”-type strategy is present only for that strategy—S—that do not actively forage, or that forage in a random fashion—RF.

The two highest accumulation levels, 55 and 105, appear to have identical behaviours when using a fixed strategy within an environment with a fixed probability of regeneration. This effect is a consequence of the parameter $E_s$: For an environment where $E_s = 2.0$, and $M_b = 0.05$, agents cannot exceed $E_{\alpha }(t) = 40$, thereby explaining why agents with levels 55 and 105 have identical behavior (Fig. 1). In order for an accumulation level $>55$ to be useful it is necessary to overeat sufficiently to go beyond this energy level. To make this possible, we increased $E_s$ to 3.0.

**Figure 2:**
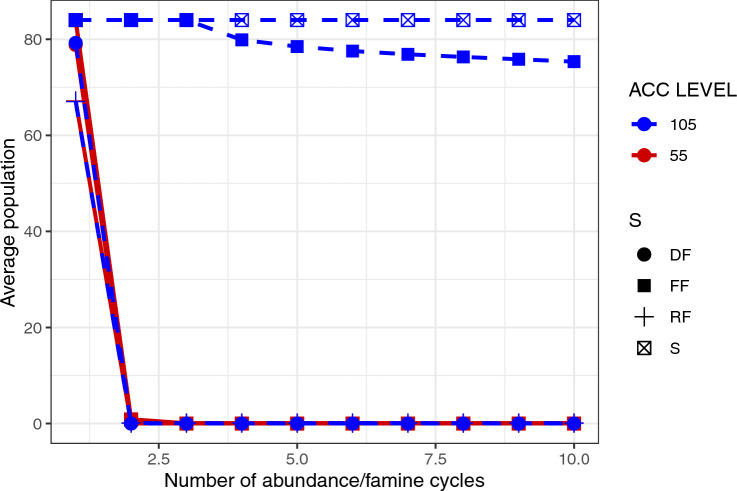
The average population during a particular cycle for S, RF, DF and FF agents using repetition as heuristic with accumulation levels of 55 and 105. In these simulations, agents experience a period when food is regenerated immediately after being eaten, followed by a period where food is not regenerated. These periods are fixed to 60 and 40 time steps respectively. The average per cycle considers both periods and the 30 repetitions of every set of parameters and error bars on the y axis represent the standard deviation of the ensemble. Graphs are slightly displaced on the x-axis for visualization.

**Figure 3:**
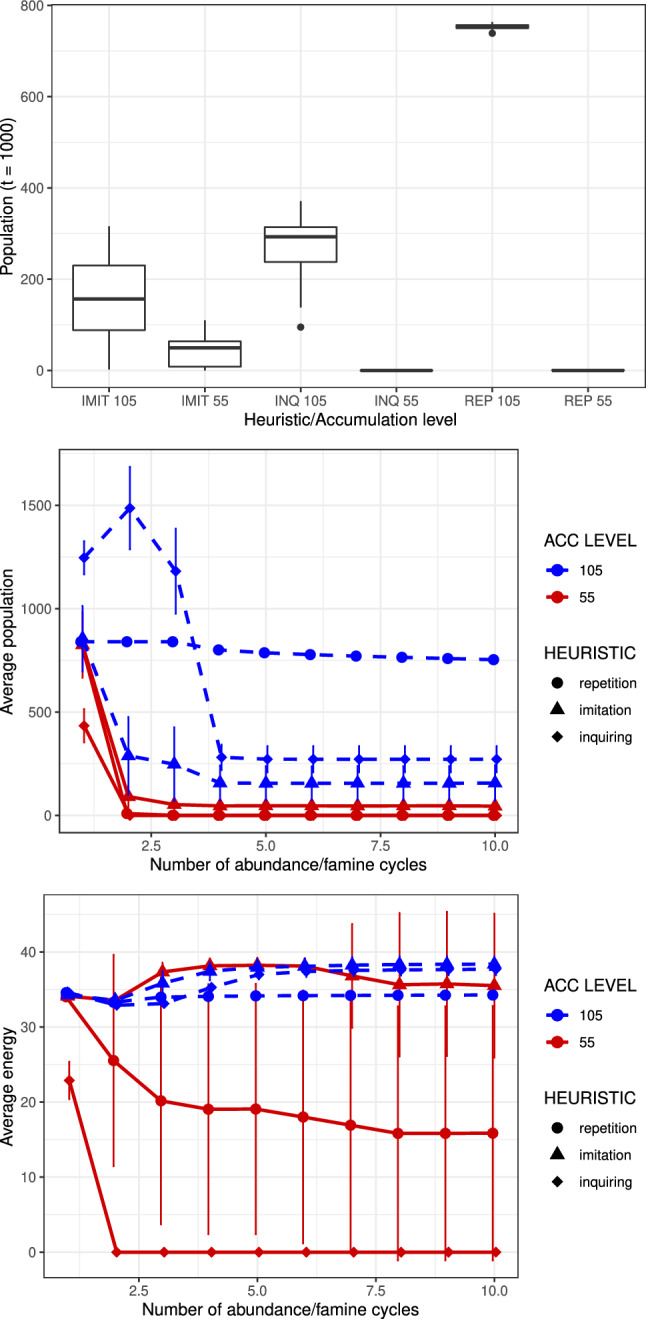
Top: Box plots of the population of agents with accumulation levels 55 and 105, at time $t = 1000$ when foraging strategy FF is used and the agent’s accumulation level is potentially subject to adaptive change according to one of three heuristics: repetition, imitation and inquiring. Comparison through a set of “energy availability cycles” of: Middle: the average population and Bottom: the average energy during a particular cycle for FF agents with accumulation levels of 55 and 105. In these simulations, agents experience a period when food is regenerated immediately after being eaten, followed by a period where food is not regenerated. These periods are fixed to 60 and 40 time steps respectively. The average per cycle considers both periods and the 30 repetitions of every set of parameters and error bars on the y axis represent the standard deviation of the ensemble. Graphs are slightly displaced on the x-axis for visualization.

The results of the second set of experiments can be seen in Figs. 2 and 3. In Fig. 2 we see the relative advantage of the more thrifty 105 accumulation level versus the 55 level for agents that use the same foraging and consumption strategy. In this case only the sedentary, S, or potentially sedentary, FF, strategies survive. In Fig. 3, we restrict attention to the FF strategy. In the Top graph, we show box plots of populations of FF agents associated with the last time step after completing 10 abundance-scarcity cycles for different decision heuristics. We can observe that agents with energy level 55 end with an average population of around 43 for imitation, but close to zero for the repetition and inquiring heuristics. When the accumulation level is 105, however, the average population is greater than 100 for every considered heuristic. In other words, survival probability is significantly enhanced for the higher energy accumulation level agents.

In Fig. 3 we see the population average (middle graph) and the average energy per agent (bottom graph) throughout 10 abundance-scarcity cycles for different heuristics and different initial accumulation levels, where the averages are computed by considering the abundance phase of each cycle i.e., averaging values of every time step of a given period of abundance or scarcity. We see that, for each heuristic, the population average is greater for the higher accumulation level agents throughout the set of abundance-scarcity cycles, although there are significant differences between the heuristics. Note that level 55 agents exhibit steep, monotonic declines after the first cycle, with the decrease being particularly notable for the repetition and inquiring heuristics. The behaviour of the level 105 agents with the inquiring heuristic is quite noticeable, with a robust increase between the first and second cycles, followed by a very sharp decline between the second and fourth cycles. The imitation heuristic shows a sharp decline from the first to second cycles, followed by a more gradual decline from the second to the fourth. For both the inquiring and imitation heuristics the population average after the fourth cycle is constant. Finally, for the repetition heuristic, the population exhibits a subtle decrease after the third cycle, finishing with an average around 750 after 10 cycles.

Turning now to the average energy, for the level 55 agents, for the inquiring heuristic, the average energy goes to zero after the first cycle, while for the repetition heuristic it decreases gradually from 35 to 16 over the full set of cycles. Interestingly, for the imitation heuristic it increases slightly after the second cycle, actually exceeding that of the level 105 agents for a couple of cycles. For the latter agents, the average energy is relatively constant throughout the set of cycles, $\sim 35-40$. However, the ordering of the average energy as a function of heuristic is opposite to the ordering of the population average: repetition, inquiring and imitation for the latter and imitation, inquiring and repetition for the former.

**Figure 4:**
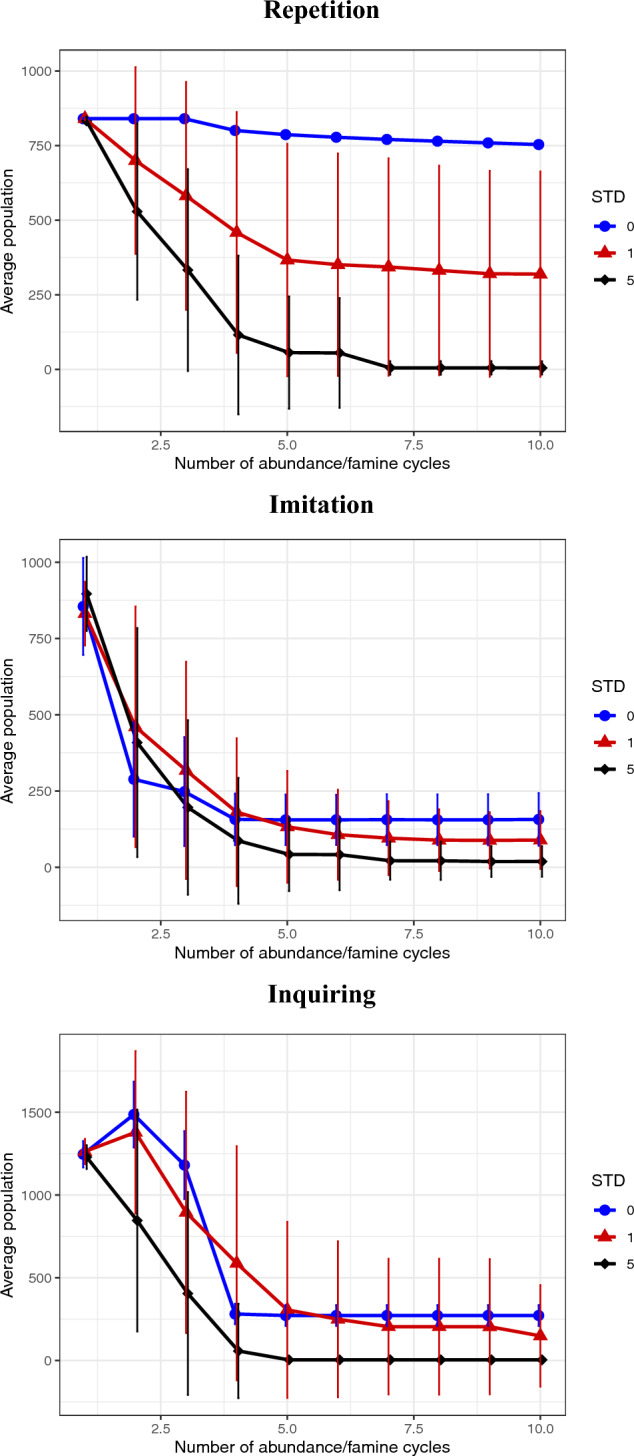
Comparison through a set of 10 “energy availability cycles” of uncertain duration of the average population at a particular cycle for FF agents with an accumulation level of 105 for systems where the employed heuristic is; Top: repetition, Middle: imitation; or Bottom: inquiring. In these simulations, agents experience a period when food is regenerated immediately after being eaten, followed by a period where food is not regenerated. The periods are chosen from a normal distribution with means 60 and 40 respectively, and with a standard deviation of 0, 1 or 5. The average per cycle considers both periods and the 30 repetitions of every set of parameters and error bars on the y axis represent the standard deviation of the ensemble. Graphs are slightly displaced on the x-axis for easier visualization.

The introduction of uncertainty in the duration of the abundance-scarcity periods produces different results according to the decision rule followed by the agents. We can clearly see that extra uncertainty has an important detrimental effect, independently of the heuristic used, with average populations decreasing as the uncertainty (STD) increases (Fig. 4). However, this ordering of population sizes as a function of uncertainty is not uniformly present as a function of time but, rather, emerges. Indeed, there is an interesting transient behaviour, such that a higher uncertainty can lead to higher average populations in the initial cycles in the case of the imitation and inquiring heuristics.

Uncertainty in the resource environment leads to a great deal of variability in population size. For repetition, uncertainty in the availability of energy resources greatly decreases the population size of the superior level-105 agents that can be maintained, with more than 50% of the agents dying across the cycle for STD 1 and almost total extinction for STD 5. This harks to the very delicate energy balance in place, where agents can just about make it through a famine that lasts 40 time steps but a famine that lasts longer can easily lead to death. If a famine lasts less than the mean however, the positive consequences—the accumulation of a bit more energy—are minimal compared to death. From an evolutionary perspective there is a strong truncation selection in play.

In the case of imitation, we see that the effect on the population average of the extra variability associated with the availability of energy resources is masked by the intrinsic variability inherent in the mistakes made by the level-105 agents that imitate the level-55 agents. If the abundance period lasts longer, this is of no significant benefit as the level-105 agents reach an energy accumulation level wherein their consumption and their metabolic needs are equal. On the other hand, as emphasized, a longer famine period easily leads to death. This is a “gambler’s ruin” type effect.

For the inquiring heuristic, the average population with STD = 1 exhibits a less catastrophic collapse than the STD = 0 population, in that the uncertainty dilutes to some degree the competition between the agents. Additionally, the inquiring agents in the STD = 1 scenarios have the possibility of storing a bit of extra energy during those periods of abundance that last longer than 60 periods.

**Figure 5:**
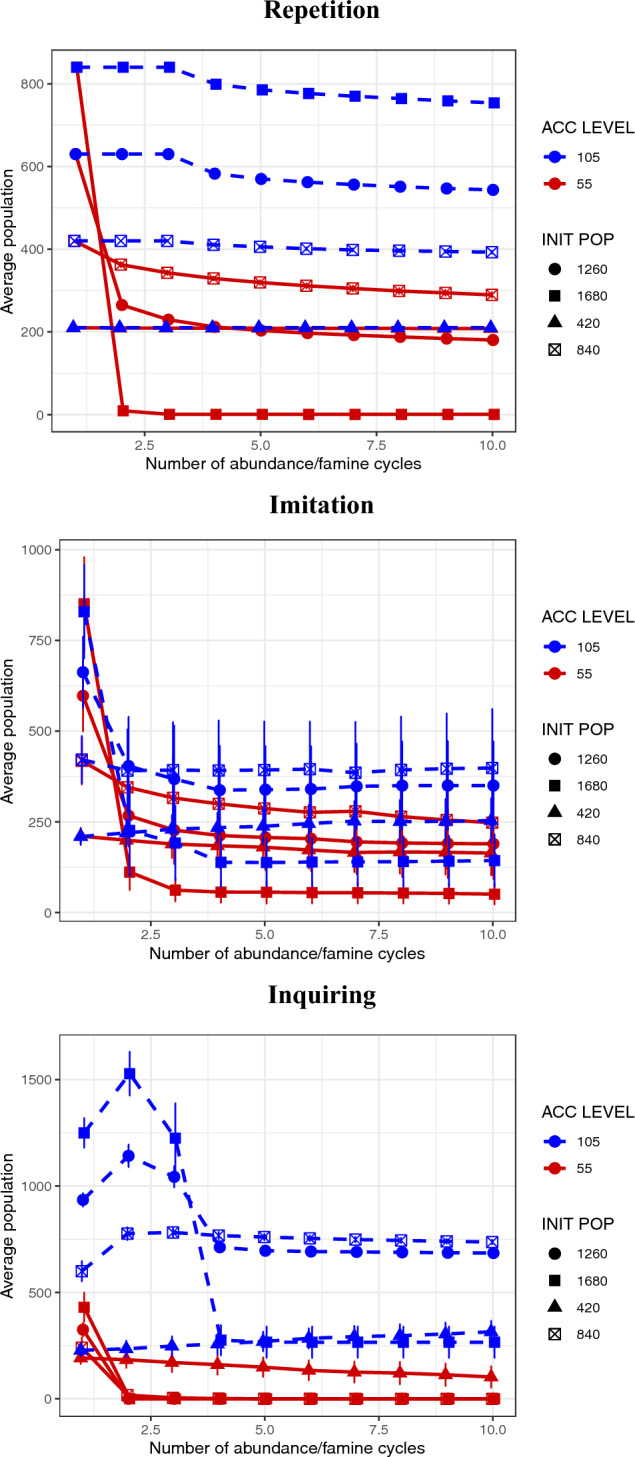
Comparison through a set of 10 “energy availability cycles” of uncertain duration of the average population at a particular cycle for FF agents with an accumulation level of 55 or 105, and for an initial population of 1680, 1260, 840 and 420 agents, for systems where the employed heuristic is; Top: repetition, Middle: imitation; or Bottom: inquiring. In these simulations, agents experience a period when food is regenerated immediately after being eaten, followed by a period where food is not regenerated. These periods are fixed to 60 and 40 time steps respectively. The average per cycle considers both periods and the 30 repetitions of every set of parameters and error bars on the y axis represent the standard deviation of the ensemble. Graphs are slightly displaced on the x-axis for easier visualization.

Finally, in Fig. 5 we see the effect of initial population size in the context of simulations with an FF strategy and different heuristics. Although the relative benefit of the higher accumulation level remains, its magnitude is seen to depend on both the initial population and the heuristic used, with the dependence on the former being non-linear for the imitation and inquiring heuristics. Additionally, we see that the prominent collapse of the population for the inquiring heuristic is reduced and eventually disappears as the initial population decreases.

## Discussion

The results of the experiments seen in Figs. 1, 2 and 3 exhibit under what circumstances a particular energy storage strategy has an advantage over others. Figure 1 shows that there is a slight advantage associated with a higher capacity, or willingness, to store energy—a “thrifty gene” behaviour—in environments with scarce food resources, but only for strategies S and RF. However, this is an extreme case of a more general trait—that there is no point having a thrifty gene if there does not exist another fundamental and necessary characteristic: that agents can accumulate energy beyond their immediate metabolic needs. This, in turn, has two requirements: first, there must be enough energy resources present in the environment to make it possible to accumulate energy in the first place; and, second, the agents must behave so as to consume more than their immediate metabolic needs.

So, what is the origin of the small advantage for S and RF and why is there no advantage for DF and FF? In the case of S the relative advantage accrues from the fact that, by chance, some agents manage to consume a higher than average amount of food resources in a given cell, so that the storage of this extra energy allows them to survive in that cell when there is less food than expected. In other words, the uncertainty inherent in the distribution of food resources that can lead to agents dying, by “bad luck”, can be partially offset by an energy storage capacity that is utilized by those agents that, by “good luck”, manage to obtain more food resources than the average. Again, though, this requires sufficient energy resources and the tendency to eat beyond their immediate metabolic needs. For RF, which represents a Brownian walk^44–46^, the advantage is reduced relative to S because the agents have an extra expense due to movement. For DF this expense is even greater, due to perception, while in this food-scarce environment FF is effectively the same as DF, as the lack of food will cause the agents to always be on the move. The fact that for S there is an advantage for levels 105, 55 and 15 relative to 6, while for RF they are all equally better than the level 5 baseline speaks to the fact that the agents on average are unable to accumulate energy $> 15$ units in the former case and $> 6$ in the latter. These results indicate how subtle the interplay between energy consumption and expense can be, even in this simple case.

It is interesting to note that in the case of very scarce resources—$p<=0.01$—the advantage disappears. This is due to the fact that the probability of accumulating sufficient energy above the baseline level of 5 is negligible. In other words, energy storage is of no use if food is so scarce that it is highly improbable to obtain enough energy resources to take advantage of it. On the contrary, above a certain $p_g$, energy storage is not even necessary, as there are always sufficient food resources available.

If greater energy storage capacity offers no significant advantage in resource environments that are constant in time, then under what circumstances might it be useful? The results seen in Figs. 3 and 4 clearly answer this question: Firstly, in those environments where periods of relative scarcity and relative abundance alternate; and, secondly, in those environments that have some degree of regularity in the duration of the feast-famine cycles, with the relative advantage depending on the foraging and consumption strategy used, as well as the precise energy storage capacity relative to the duration of the periods of abundance and famine. Agents that forage can potentially survive during the famine, in spite of their extra energy cost, by finding any unconsumed food resources that are left over from the abundance period when the famine began. Of course, such an agent must also survive the competition from its peers in the search for these unconsumed resources.

The results for systems with fixed periods of abundance and scarcity confirm the survival of S (as the system parameters have been chosen to make them do so). We can think of this as the “hibernating bear” scenario, wherein the advantage of energy accumulation accrues only if the agent maintains the lowest energy expenditure possible. Apart from S, only the FF strategy is able to support large populations of agents that have the higher accumulation level. The other strategies all have to deal with extra energy costs, and, in a time of scarcity, this places a great deal of extra selection pressure. FF agents, however, can regulate their expenditure of energy based on their perception of the environment, remaining static (“sedentary”) during the abundance periods as the resource regeneration guarantees the existence of food in the next time step. In the periods of scarcity, every amount of saved energy is vital. In this case, rather than a “thrifty” behaviour as being of relevance, we may speak of the possibility of a sedentary behaviour, that suppresses physical activity in circumstances where active foraging is not advantageous. Of course, this is not to say that active foraging is never useful. The capacity to adapt foraging strategy to a predictable spatial distribution of food can result in a survival advantage^44,47^.

Thus, higher energy storage alone is not sufficient to survive. Rather, it is the intelligent balance between consumption in place versus movement that permits the higher accumulation level to exhibit an advantage. More generally, we see that the benefit of energy accumulation levels—the “thrifty genotype”—is dependent on the foraging strategy and heuristic used by the agent relative to the temporal availability of food and the competition from other agents. Of course, as emphasized, a necessary condition for taking advantage of exploiting a higher accumulation level is also the potential to keep consuming in order to reach that level above and beyond the short-term energy needs of the agent.

The existence of heuristics introduces an extra level of complexity by permitting agents to change their energy accumulation strategy in the case of imitation and inquiring, with the difference between them being that agents using the imitation heuristic simply copy at random an energy accumulation strategy in their community, whereas an agent using the inquiring strategy will always choose the best one in that community (in this case, the largest accumulation level). With repetition, there is no possibility for a level-55 agent to learn or copy the level-105 strategy, and therefore the mortality of the level-55 agents is almost total.

The heuristic does not determine the level of benefit of energy accumulation but affects the dynamics of competition between agents (Fig. 4). The repetition heuristic for the level-105 agents is the most successful because it avoids both the mistakes associated with the imitation heuristic and the excessive level of initial success of the agents with the inquiring heuristic. For the imitation heuristic, level-105 agents may copy level-55 agents, as well as vice versa, and, in this case, those level-105 agents that have switched are more at risk. However, there is a bias, in that during the famine the level-55 agents will have a higher mortality rate and therefore there will be less of them to imitate, thus leading to a relative excess of level-105 agents. For the inquiring heuristic, many of the 840 original level-55 agents have imitated the superior level-105 strategy. Those that don’t - die. The case that there is a collapse in the level-105 population after the second cycle is due to the fact that they are victims of their own success, with the resources available during the scarcity periods not sufficient to support such a large population. Agents with the 55-level adopt the 105-level as soon these agents can accumulate more than 55, and this trait spreads rapidly enough to dominate the population before the famine. With this strong presence of competing agents, frequently coinciding in the same positions, many agents die after the sudden peak of population. Fig. 4 also shows the potentially devastating affect of unpredictable variation in the food distribution, where for STD=5 the highest accumulation level cannot survive independently of the heuristic used. This is a consequence of a period of scarcity that just lasts too long for the maximum acquired energy. Finally, in Fig. 5 the differences due to initial population size show how the latter affects the degree of competition between agents and also the notion of just how scarce resources become in the scarcity periods. The lower the population density the more resources are left over at the end of the abundance periods and therefore the less the competitive advantage of the higher accumulation level agents. Similarly, the collapse in population in the case of the inquiring agents with level 105 is reduced, as the degree of intra-specific competition is now less.

Two important characteristics of the obesity epidemic are its ubiquity and its resilience. With respect to ubiquity: What differs between one country and another is not whether there is a problem with overweightedness and obesity, and its concomitant health problems, but, rather, just how severe the problem is. Effectively, up to now only some sub-Saharan countries have avoided the problem. With respect to resilience, it exists at both the individual level and the group level, where, at the individual level, reversion of the obese state to normal weight is very difficult^48^, while, at the group level, it has been exceedingly difficult to design public health policies that have both a significant impact and are widely adopted by the population.

Two potential, complementary explanations for the ubiquity of the obesity epidemic are genetics, such as the thrifty genotype hypothesis, and the recent development of an obesogenic environment. Thrifty genotype explanations blame our genetic heritage, but in a causally indirect way, in that the purported genes are associated with physiological adaptations that make it easier to get fat. On the other hand, blaming the environment seems to neglect the fact that we ourselves are the creators of that environment. It is undeniable that widespread obesity was not a problem for our prehistoric ancestors, but that it is now. But how do you test hypotheses about such changes? ABM are one promising avenue for creating and testing such hypotheses. If we take our energy storage parameter as a proxy for a “physiological” thrifty gene, we have shown that it can offer a selective advantage in the context of resource environments that exhibit seasonal variability, as is common in many traditional societies and was probably true for our early ancestors, with the abundance period being essential in order to store up energy in the first place. However, we have also shown that there must be present two important behaviour types - conducts - in order for extra energy storage to be a useful adaptation: consumption over expenditure and sedentariness. By sedentariness, we mean that energy expenditure by unnecessary activity is selected against. This is manifest in the success of the FF strategy, where movement is initiated only if there is no food resource in the agent’s cell but there is in an adjacent cell.

As our results indicate that there is a selective advantage in higher accumulation and sedentariness in energy environments that have periods of abundance and scarcity and, more generally, uncertainty, we must ask how might this selective advantage have manifested itself? Such a strong selective effect must surely have induced, depending on the modeled timescale, a genetic or cultural response, and therefore, subsequently, left a strong genetic or cultural imprint. We believe that the legacy of an imprinted tendency to overconsume and be sedentary, when combined with a “thrifty gene” physiological response that gives the opportunity to store the excess energy, led to the current obesity epidemic. Indeed, our simulation of an energy rich environment after a period of selection through abundance-scarcity cycles clearly shows that agents reach their maximum energy storage through the twin effects of overconsumption and sedentariness. It is here again that the question of what the energy accumulation level represents enters. We argue that taken as a “normal” level, in evolutionary terms it represents the tuned balance between the benefit of energy accumulation in situations of food scarcity with the, here implicit, costs of excess adiposity. The more constant the food environment the less the need for energy storage. However, given that, presumably, our normal levels of fat of  10-25% are an adaptation to past food environments and, as we have shown, that overconsumption and sedentariness were necessary behavioral corollaries in order to maintain normal fat levels, our on-demand and constant food environment, coupled with the previously adaptive overconsumption and sedentariness behaviors, has led to fat accumulation beyond this normal level.

Although there is much future work to be done, we can see the subtle complexity that enters when adding in heuristics which, here, represent variation, and can be thought of as analogs of mutation or learning. Heuristics change the competition between agents, including those of the same type. These changes can temporarily exacerbate the advantage of accumulating behaviors. This is evident, for example, when on a short time scale it is much better for a population of these agents to use an inquiring heuristic, while, in contrast, in the long term it is better to use a repetition heuristic.

Finally, it is important to indicate the limitations of this study. Obesity is a consequence of long term imbalance between energy expenditure and energy consumption, both of which are immensely complex. We have made enormous simplifications while, we hope, capturing important elements, such as basal metabolism and the costs of foraging on the energy expense side, and overconsumption on the other side. Our foraging strategies are simple relative to the complexity of those that exist in humans or, indeed, most animals. Similarly, we have also considered only a particular set of food environments that are controlled by one parameter, $p_g$ and concentrated on changes in the temporal as opposed to spatial variability. There is a great deal of literature, both theoretical and observational (see for example^45^ ), that considers the subtle interplay between which foraging strategies, e.g., Brownian motion versus Levy walks, are associated with which food environments, e.g., random and dense versus patchy. Thus, as well as exploring more fully the parameter space of the current ABM, there is huge scope for including new parameters. The simplicity of our agents implies that they do not capture the complexity of the evolution of human behavior. However, the simplicity is such that we believe that our results are applicable to any “creatures” that have to survive in environments with strong seasonal variation in food availability.

## Data Availability

The datasets generated during and/or analysed during the current study are available in the ABM Heuristic Decision-Making Energy repository, https://figshare.com/projects/ABM_Heuristic_Decision-Making_Energy/121542.
